# The efficacy of dermoscopy in defining the surgical margins of cutaneous squamous cell carcinoma: a retrospective study

**DOI:** 10.3389/fonc.2023.1141820

**Published:** 2023-04-28

**Authors:** Zhenru Liu, Shudai Huang, Fang Li, Xiaoqing Wang, Mengxi Liu, Hoi Shiwn Wong, Jiayi Jiang, Yuan Zhou, Daguang Wang

**Affiliations:** ^1^ Department of Dermatology, The First Affiliated Hospital with Nanjing Medical University, Nanjing, China; ^2^ Department of Dermatology, Jen Ching Memorial Hospital, Suzhou, China; ^3^ Department of Plastic & Cosmetic Surgery, Women’s Hospital of Nanjing Medical University (Nanjing Maternity and Child Health Care Hospital), Nanjing, China; ^4^ Department of Dermatology, Suzhou Municipal Hospital East Area, The Affiliated Suzhou Hospital of Nanjing Medical University, Suzhou, China

**Keywords:** cutaneous squamous cell carcinoma, dermoscopy, incisional biopsy, excisional biopsy, surgical margin

## Abstract

**Objective:**

To investigate the diagnostic value of dermoscopy in defining the tumor margin of cutaneous squamous cell carcinoma (cSCC) for the appropriate surgical margin.

**Methods:**

A total of 90 cSCC patients were enrolled in the study. All patients were recruited into two groups: those who preserved intact macroscopic features of neoplasms without or after incisional biopsy and those with uncertain residual tumors after excisional biopsy. A dermoscopy-defined surgical margin of 8mm outward was used according to the tumor boundaries observed with the naked eye and dermoscopy. All excised tumor specimens were divided into serial sections according to the four “3, 6, 9, 12” directions at every 4-mm interval from the dermoscopy-detected tumor margin. Pathological examination was performed at 0 mm, 4 mm, and 8 mm margins to confirm tumor remnants.

**Results:**

Retrospective analysis of dermatoscopic results showed inconsistent clinical and dermatoscopic borders in 43 of 90 cases (47.8%). The ability of dermoscopy to detect tumor borders showed no statistical difference between the two groups (p > 0.05). In the unbiopsy or incisional biopsy group, 66.6% of the tumors were resected with a 4-mm margin and 98.3% with an 8-mm margin, with significant differences (p = 0.047). For patients with inconspicuous clinical evidence of residual tumor after excisional biopsy, the tumor clearance rate was 53.3% at 0 mm, 93.3% at 4 mm, and 100.0% at 8 mm. Statistically significant differences were noted between 0 mm and 4 mm (p = 0.017), as well as between 0 mm and 8 mm (p = 0.043) but did not differ between 4 mm and 8 mm (p > 0.05).

**Conclusions:**

Dermoscopy defined the tumor margin of cSCC better than visual inspection alone. Direct dermoscopic-guided surgery with at least 8-mm expansion was recommended for high-risk cSCC. Dermoscopy also assisted in identifying surgical margins at the healing biopsy site, making 8 mm still the recommended expansion range.

## Introduction

Cutaneous squamous cell carcinoma (cSCC) is the second most common non-melanoma skin cancer (NMSC), with a rising incidence in recent decades (1). Sun exposure, age, fair skin, and immunosuppression are the most significant risk factors associated with cSCC development. The appearance of lesions in cSCC is not easily distinguished from other NMSCs, and biopsy helps confirm the diagnosis and histopathological subtype. In cases of inadequate micro-staging indicated during incisional biopsy, excisional biopsy may be considered (2).

Although most cSCC patients can be cured with local treatment, some high-risk cases present more aggressively with poor prognoses. Complete tumor excision and clear histological margins are critical to treatment. The European Organization for Research and Treatment of Cancer (EDF-EADO-EORTC) guidelines stated a minimal standardized margin of 5 mm for low-risk cSCC (lrSCC) and 6-10 mm for high-risk cSCC (hrSCC) (3, 4). The National Comprehensive Cancer Network (NCCN) Guidelines recommend a 4-6mm resection margin for lrSCC (2). Due to wide variability in clinical characteristics, it is considered infeasible to recommend the defined margin for hrSCC (2). For increased safety, Schell (5) indicated that 13.25 mm margins were required to achieve a 95% clearance rate in hrSCC, which is unreasonable in most patients. A wide local excision (WLE) will lead to poor aesthetics or impaired function of facial features since hrSCC commonly originates in the head and neck. And patients may present unascertainable residual tumors after excisional biopsy, requiring further surgical management without such extensive margins. Mohs micrographic surgery (MMS) is considered the most effective treatment (6, 7). Pathological examination of the specimen intraoperatively ensures a clean surgical edge and maximum preservation of normal tissue. However, the widespread use of MMS is limited given its time consumption, high labor costs, equipment requirements, and the need for cooperation from dermatologists (8).

In recent years, dermoscopy has been recommended as a user-friendly, relatively inexpensive, non-invasive technique that provides a basis for diagnosis by observing epidermal structures, pigments, blood vessels, and skin appendages (9, 10). The dermoscopic features of cSCC are described as polymorphic vascular patterns with scales or keratinous crusts in the central area. Recent studies have confirmed that presurgical dermoscopic analysis is more valuable than clinical observation in determining tumor margins (11, 12). The goals of our study are to ascertain the significance of dermoscopy in evaluating surgical margins for both cSCC and the unascertainable residual tumors after excisional biopsy. Additionally, we attempted to ascertain the margin distance for dermoscopy-guided surgery as a secondary goal.

## Materials and methods

### Patients

This study was approved by the Ethics Committee of the First Affiliated Hospital with Nanjing Medical University, and each patient signed the informed consent form. A total of 90 patients with cSCC who visited the Department of Dermatology, the First Affiliated Hospital with Nanjing Medical University, from May 2016 to March 2022 were recruited as research objects. The location, size and grade of differentiation of cSCC were classified according to the criteria outlined in the NCCN Guidelines (2).

### Inclusion criteria and exclusion criteria

Patients with cSCC were recruited into two groups: those with intact tumor morphology without biopsy or after incisional biopsy, and those with inconspicuous clinical evidence of residual tumor after complete incisional biopsy. We excluded patients with other skin lesions that interfered with diagnosis.

### Surgical tissue processing

**Figure 1:**
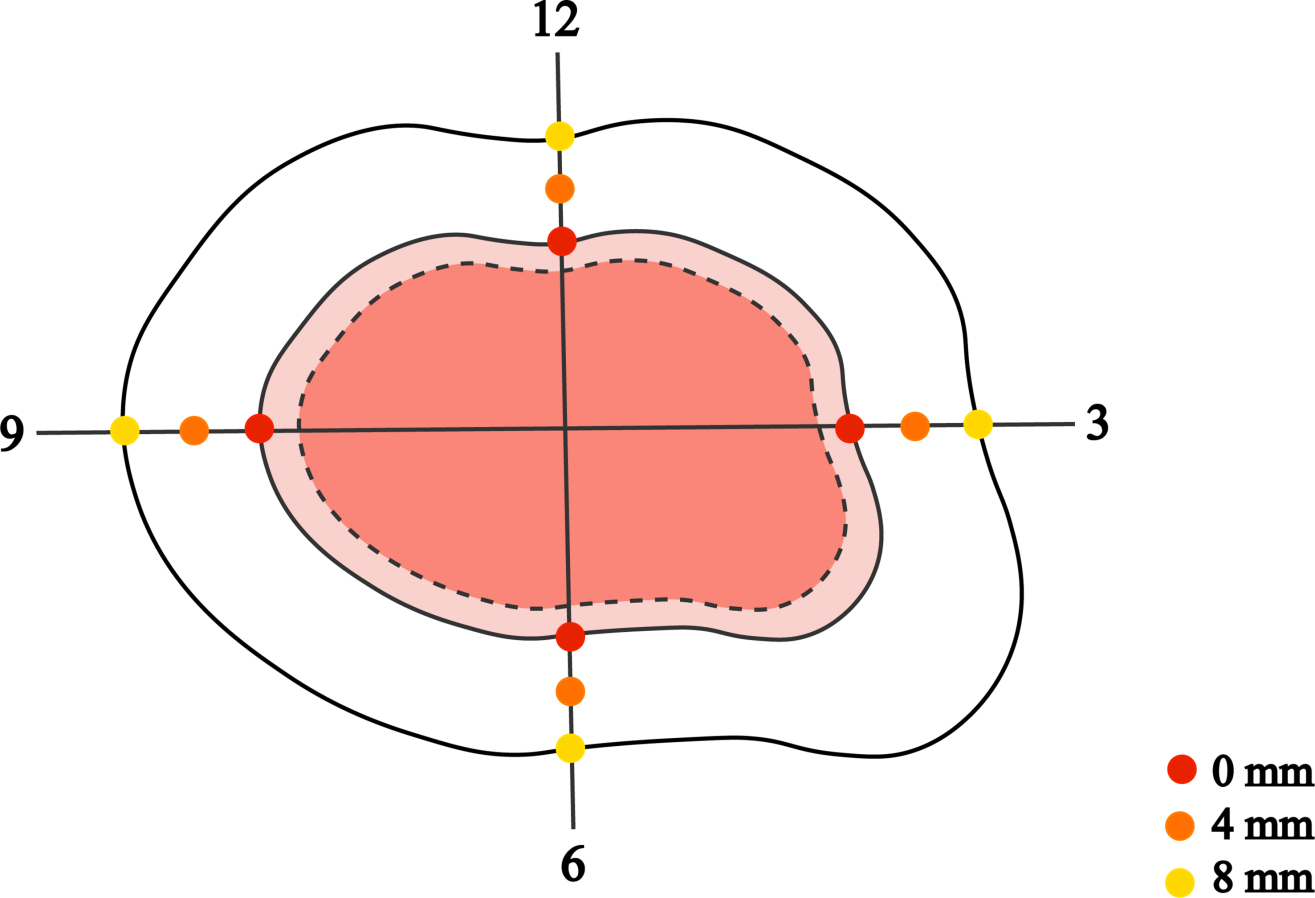
The dashed and solid lines mark the tumor boundary as determined by the naked eye and by dermatoscopic examination, with the dermatoscopic boundary 8 mm outwards for the surgical margin. All excised tumor specimens were divided into serial sections according to the four “3, 6, 9, 12” directions at every 4-mm interval from the dermoscopy-detected tumor margin. Pathological examination was performed at 0 mm, 4 mm, and 8 mm margins to confirm tumor remnants.

Visual examination and palpation preliminarily determined the tumor margin. Four of the “3, 6, 9, 12” directions and the naked-eye boundaries were marked with dotted lines using a demographic pencil (Tondaus, T3023, Germany). Then, the margins were corrected with digital dermatoscopy (Dermlite dl3, 3Gen, USA) and recorded with solid lines. A digital camera (G16, Canon, Japan) took clinical and dermoscopic images. All the lesions were surgically excised along the superficial cut extending 8mm outwards from the dermoscopy-marker margin. Superficial tumors and tumors with smaller diameters were resected to the deep layer of subcutaneous fat. Larger and deeper tumors were excised to the surface of muscle, cartilage, or periosteum. The tumor was resected completely along the preoperatively determined cutting edge, and a skin graft or flap was used to cover the skin defect to promote primary healing. Postoperatively, the laterally enlarged margin was selected from the four “3, 6, 9, 12” directions and submitted to a uniform method of histological examination with serial-parallel sections at 4-mm intervals (**Figure 1**).

### Follow-up

All patients were followed up regularly, every three months for the first year after surgery, every six months for the second year, and annually after the third year.

### Statistical analysis

Categorical data were described by absolute and relative frequency (%), while continuous data by mean and standard deviation. Age, sex, location, and recurrence were analyzed using chi-square to confirm associations between groups. Pathological findings were compared among patients with different excision margins. Kendall’s test was conducted for multiple group comparisons with a Bonferroni statistic for *post-hoc* analysis. A p-value of less than 0.05 indicated statistical significance in all analyses.

## Results

### Patient characteristics

The study involved 90 patients with histologically confirmed cSCC, 51 (56.7%) of whom were male and 40 (44.4%) female (**Table 1**). The age range of the patients was 52 to 93; the average age of the sample was 74.0 years. The most common region was in the H area (52.2%) and 86 (95.6%) patients were diagnosed as well differentiated. All patients were classified as high-risk or very-high-risk cSCC according to the latest NCCN Guidelines. Out of the total patients, 5 patients (5.6%) with recurrence and 1 patient (1.1%) with metastasis. There was no notable discrepancy in the recurrence or metastasis rate between the two patient groups, and no correlation was found between the prognosis and demographic characteristics or clinical manifestations.

**Table 1:** Demographics and tumor characteristics.

	Total (n = 90)	Unbiopsy or incisional biopsy (n = 60)	Excisional biopsy (n = 30)	P
Age, years (mean [SD])	74.0 (10.6)	75.5 (10.6)	71.2 (10.2)	0.197
Male (%)	51 (56.7)	38 (63.3)	13 (43.3)	0.071
Location^*^ (%)				0.113
Area H	47 (52.2)	36 (60.0)	11 (36.7)	
Area M	34 (37.8)	19 (31.7)	15 (50.0)	
Area L	9 (10.0)	5 (8.3)	4 (13.3)	
Grade of differentiation (%)				0.508
Well differentiated	86 (95.6)	58 (96.7)	28 (93.3)	
Moderately differentiated	3 (3.3)	2 (3.3)	1 (3.3)	
Poorly differentiated	1 (1.1)	0 (0.0)	1 (3.3)	
Recurrence (%)	5 (5.6)	5 (8.3)	0 (0.0)	0.165
Metastasis (%)	1 (1.1)	1 (1.7)	0 (0.0)	1.000

^*^ Location: According to the risk of different skin lesions in the NCCN Guidelines they are classified into three areas. Area H consists of the face (middle face, eyelids, eyebrows, periorbital, nose, lips [cutaneous and vermilion], mandible, palate, temples, ears, skin/grooves in front of and behind the ears), genitals, hands, and feet. Area M includes the cheeks, forehead, scalp, neck, and shin front. Area L includes the body and limbs (except foreskin, hands, feet, nails, and ankles)^2^.

SD, standard deviation.

### Characters of the dermoscopic manifestations

**Figure 2:**
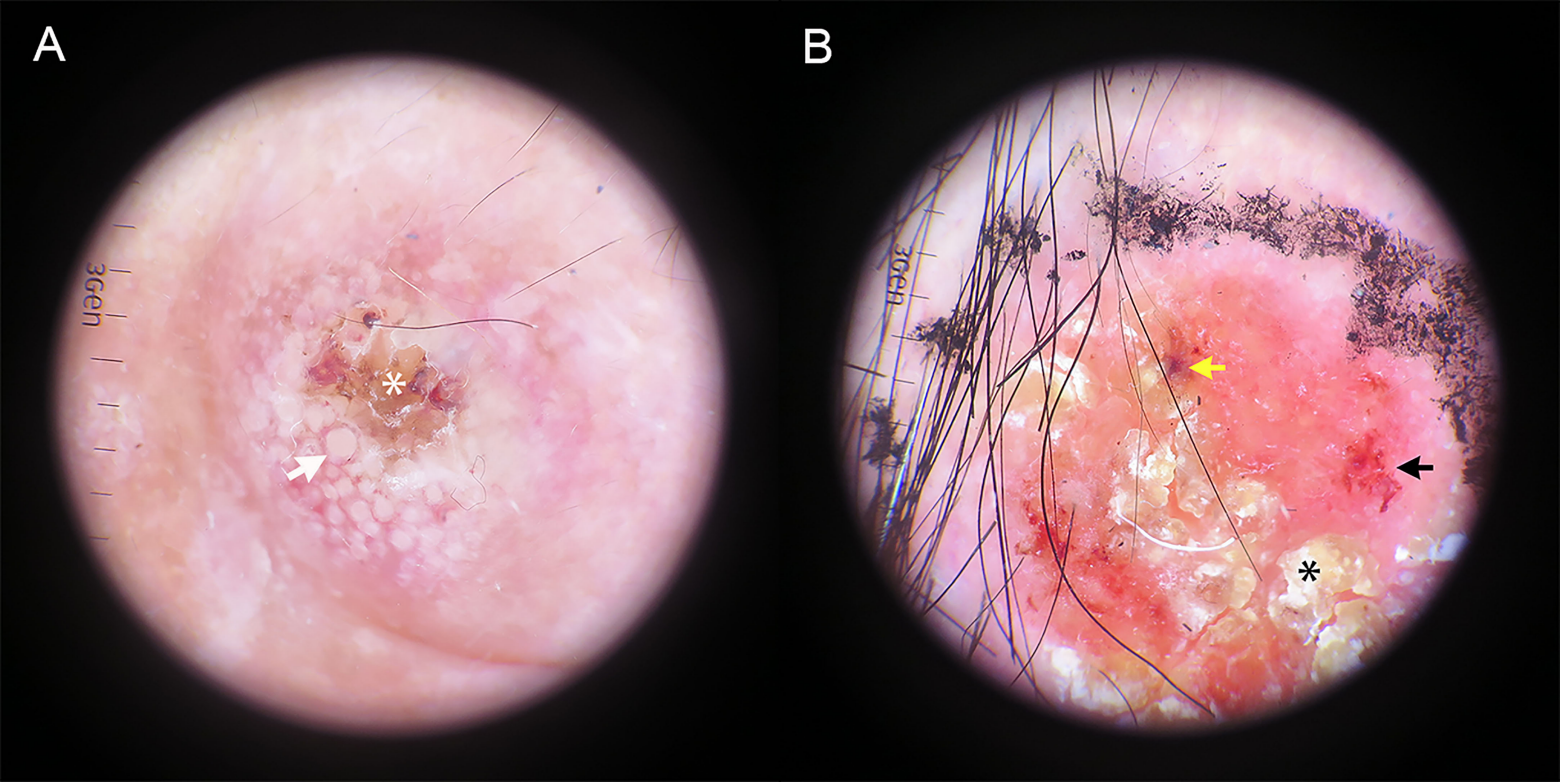
Dermatoscopic image of hrSCC without biopsy. **(A)** Targetoid-appearing hair follicle openings (white arrow) can be seen around the amorphous central keratotic area (white asterisk). **(B)** A red predominant color, yellow-white scale (black asterisk), dotted/glomerular vessels (black arrow) and blood spots (yellow arrow) are seen.

**Figure 3:**
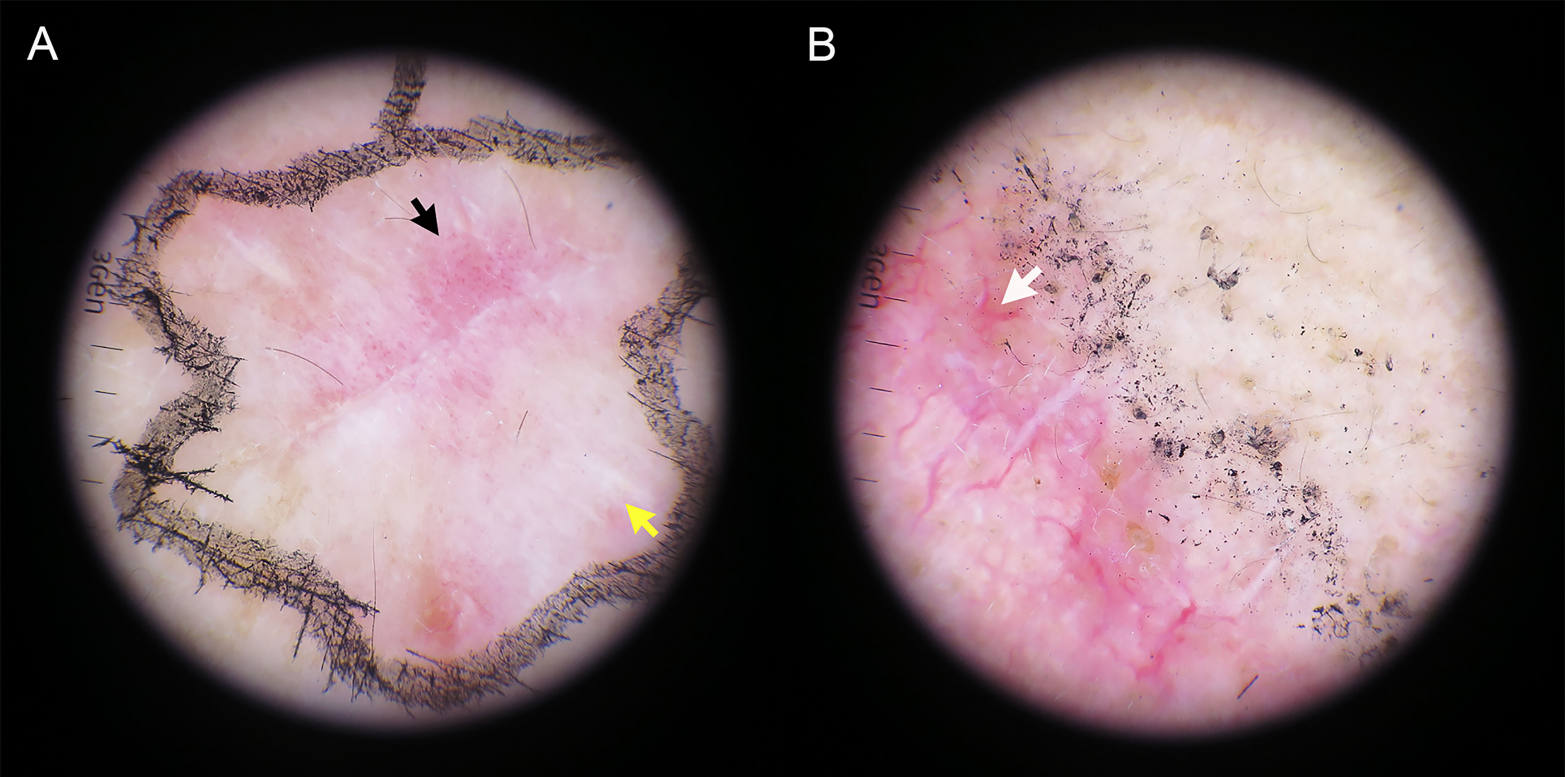
The dermatoscopic appearance of hrSCC after complete incisional biopsy. **(A)** Glomerular vessels (black arrow) coalescing with shiny white strands (yellow arrow) are seen. **(B)** A predominantly white color intertwined with a branch-like linear vessel (white arrow).

**Table 2** summarizes the total number and frequency of dermoscopic patterns. Among the classic dermoscopic patterns of cSCC, the scale exhibited the highest frequency at 65.6%, followed by polymorphic vascular patterns at 61.1%, with glomerular vessels being the most prevalent (**Figure 2**). Shiny white blotches and strands (p < 0.01) and the absence of central keratin mass (p < 0.05) were significantly more common after excisional biopsy (**Figure 3**). In univariate analysis, it was found that no dermoscopic features were able to predict recurrence, metastasis, or positive histological margins.

**Table 2:** Frequency of the dermoscopic manifestations.

	Total (n = 90)	Unbiopsy or incisional biopsy (n = 60)	Excisional biopsy (n = 30)	P
Vascular pattern				
None	2 (2.2%)	1 (2.0%)	1 (3.3%)	1.000
Monomorphous	24 (26.7%)	11 (22.0%)	13 (43.3%)	0.076
Polymorphous	55 (61.1%)	38 (76.0%)	17 (56.7%)	0.085
Vessel morphology				
Dotted/Pinpoint	11 (12.2%)	9 (18.0%)	2 (6.7%)	0.195
Glomerular	55 (61.1%)	38 (76.0%)	17 (56.7%)	0.085
Hairpin	40 (44.4%)	26 (52.0%)	14 (46.7%)	0.818
Linear-irregular	34 (37.8%)	15 (30.0%)	19 (63.3%)	0.005
Blood spots	46 (51.1%)	35 (70.0%)	11 (36.7%)	0.005
Ulcerations	21 (23.3%)	15 (30.0%)	6 (20.0%)	0.433
Scales	59 (65.6%)	40 (80.0%)	19 (63.3%)	0.121
Central keratin mass	26 (28.9%)	23 (46.0%)	3 (10.0%)	**<0.005**
Targetoid hair follicles	39 (43.3%)	23 (46.0%)	16 (53.3%)	0.645
Rosettes	21 (23.3%)	12 (24.0%)	9 (30.0%)	0.605
Keratin pearls	39 (43.3%)	23 (46.0%)	16 (53.3%)	0.645
White halos	26 (28.9%)	20 (40.0%)	6 (20.0%)	0.086
White structureless areas	48 (53.3%)	29 (58.0%)	19 (63.3%)	0.814
Shiny white blotches and strands	26 (28.9%)	2 (3.3%)	24 (80.0%)	**<0.001**
Background erythema	35 (38.9%)	19 (38.0%)	16 (53.3%)	0.245
Red pseudonetwork	32 (35.6%)	18 (36.0%)	14 (46.7%)	0.358

p<0.05 was considered statistically significant.

### Dermoscopy-guided surgical excision margin

**Figure 4:**
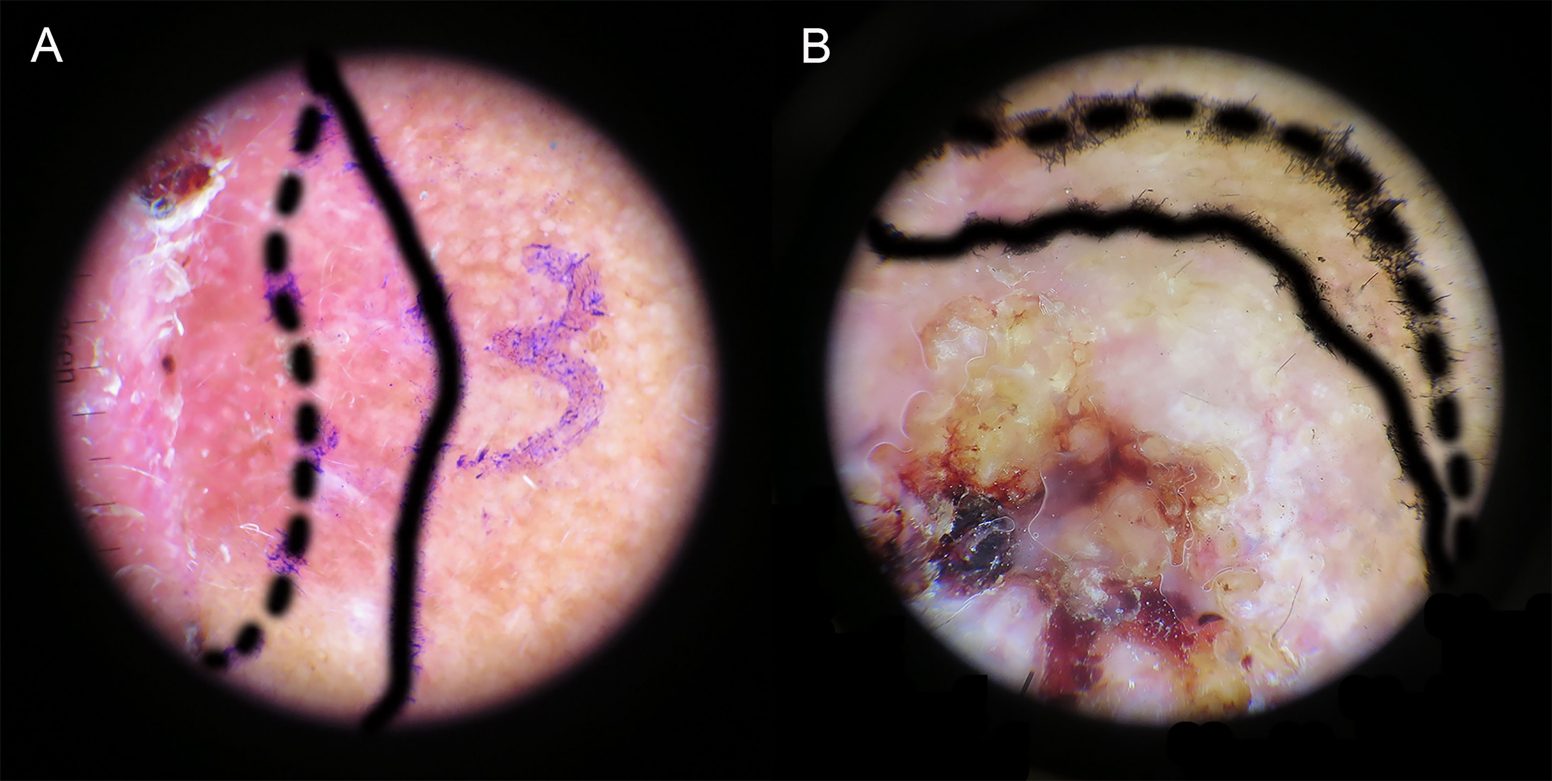
Delineation of tumor boundaries by naked-eye (dashed line) and dermoscopy (solid line). **(A)** Wider dermoscopic edge. **(B)** Narrower dermoscopic edge.

Retrospective analysis of dermatoscopic images showed inconsistent clinical and dermatoscopic borders in 43 of 90 cases (47.8%). The preoperative dermoscopic observation allowed for a more microscopic correction of margins, with 38.9% having wider and 8.9% narrower dermoscopic borders compared to visual margins (**Table 3**). Dermoscopy is superior to visual inspection for defining the cSCC margin (**Figure 4**). The ability of dermoscopy to detect tumor borders did not differ between the two groups (p > 0.05).

**Table 3:** Consistency of clinical and dermoscopic detection margin.

	Total (n = 90)	Unbiopsy or incisional biopsy (n = 60)	Excisional biopsy (n = 30)
Consistency	47 (52.2%)	29 (48.3%)	18 (60.0%)
Inconsistency	43 (47.8%)	31 (51.7%)	12 (40.0%)
Wider^§^	35 (38.9%)	26 (43.3%)	9 (30.0%)
Narrower^#^	8 (8.9%)	5 (8.3%)	3 (10%)

^§^Wider: The dermoscopic boundary is wider in extent compared to the boundary visible to naked eyes.

^#^Narrower: The dermoscopic boundary is narrower in extent compared to the boundary visible to naked eyes.

### Dermoscopy-guided surgical excision range

Tissues were taken from the four quadrants “3, 6, 9, and 12” for H&E staining to check whether there were residual tumor cells (**Table 4**). The complete resection rate increased with increasing peripheral surgical margins, and the differences between the expansion cuts at 0 mm, 4 mm, and 8 mm were highly significant in both groups of patients (p < 0.001). In the unbiopsy or incisional biopsy group, 61.6% of the tumors were resected at 0 mm and 66.6% at 4 mm, showing no statistical difference between them (p = 0.881). There was only one unilateral positive at 8mm dilation, and statistically different from the 0 mm and 4 mm groups (p = 0.002 and p = 0.047). For patients with inconspicuous clinical evidence of residual tumor after excisional biopsy, the tumor clearance rate was 53.3% at 0 mm, 93.3% at 4 mm, and 100.0% at 8 mm. Statistically significant differences were noted between 0 mm and 4 mm (p = 0.017), as well as between 0 mm and 8 mm (p = 0.043), but did not differ between 4 mm and 8 mm (p > 0.05).

**Table 4:** Dermoscopy-detected cSCC peripheral borders with 0-mm, 4-mm and 8-mm excision margins and their corresponding postoperative margins of pathology.

		(+)	(++)	(+++)	(–)	P
Unbiopsy or incisional Biopsy (n = 60)	0mm	13 (21.7%)	8 (13.3%)	2 (3.3%)	37 (61.7%)	p < 0.001
	4mm	15 (25.0%)	5 (8.3%)	0 (0.0%)	40 (66.7%)	
	8mm	1 (1.7%)	0 (0.0%)	0 (0.0%)	59 (98.3%)	
Excisional biopsy (n = 30)	0mm	11 (36.7%)	3 (10.0%)	0 (0.0%)	16 (53.3%)	p < 0.001
	4mm	1 (3.3%)	1 (3.3.%)	0 (0.0%)	28 (93.3%)	
	8mm	0 (0.0%)	0 (0.0%)	0 (0.0%)	30 (100.0%)	

(+) one of the four directions is pathologically positive; (++) two of the four directions are pathologically positive; (+++) three of the four directions are pathologically positive (–); no residual tumor is detected on four sides.

## Discussion

The prevalence of cSCC is increasing yearly and may be higher due to an aging population. Although most patients can be cured with local treatment, hrSCC has a tendency toward recurrence, local invasion, and distant metastasis (13). In clinical investigations, the prognosis is better when tumors are completely removed with a healthy margin of tissue. However, various guidelines for appropriate resection margins are controversial. Dermoscopy has been widely used as a non-invasive and convenient means of examination, providing more precise observation (9). There were few studies investigating the application of dermoscopy in the preoperative assessment of surgical margins in cSCC. A total of 90 patients who met the inclusion criteria were recruited into two groups. Our study suggests that the introduction of dermoscopy might have aided in the diagnosis of cSCC, even with uncertain residual tumors after excisional biopsy. In addition, it might have helped determine the optimal surgical margin.

There is mounting evidence suggesting that dermoscopy facilitates early detection of skin cancer in comparison to the naked eye (14, 15). Dermoscopy allows us to noninvasively predict the tumor’s histopathological nature, including the degree of differentiation (10, 16). Patients recruited for this study were mostly well- or moderately differentiated cSCC, with scales being the most common, followed by polymorphic vessel patterns and commonly revealing glomerular vessels. It also frequently exhibits blood spots, whitish structureless areas, perivascular whitish halos, keratin pearls, central keratin mass, and targetoid hair follicles, findings that are consistent with previous evidence (17, 18). Whole excisional biopsies resulted in a reduced presentation of central keratin plugs, coalescing with shiny white blotches and strands. These are thought to correspond to fibrosis of the underlying stroma. The lesions can appear on a predominantly red background surrounded by linear vessels, corresponding to the dermoscopic pattern of cSCC, and may also be confused with surgical inflammation. In addition, it is possible to mistake white structureless areas associated with large targetoid hair follicles for scar-like areas, appearing as porcelain-white areas without recognizable structures (10, 19). In univariate analysis, it was found that no dermoscopic features were able to predict recurrence, metastasis, or positive histological margins.

The growing use of dermoscopy in diagnosing and determining non-melanotic skin cancers has recently gained recognition. Besides helping with the diagnosis and preoperative determination of tumor boundaries (20, 21). Carducci (12) found that the margin positivity rate of cSCC was significantly higher in the clinical detection group (17%) than in the dermoscopic group (6%). Our study has revealed comparable results where the dermoscopic examination prior to surgery facilitated a more precise correction of tumor borders at a microscopic level. Among the two groups, 43.3% and 30.0% demonstrated wider dermoscopic borders compared to those identified visually. Comparatively, narrower margins were noted in only 8.3% and 10.0% of both groups. Although not a predictive factor, dermoscopy can effectively uncover subclinical tumor outgrowths by recognizing pertinent characteristics in healthy skin margins. It is more accurate at defining the boundary of cSCC than visual inspection alone. Furthermore, there is no difference in the capacity of dermoscopy to detect the tumor border between the two groups.

As previously described, hrSCC tends to local recurrence or metastasis, making an adequate safe margin of expansion essential (22, 23). However, various guidelines exist for establishing appropriate excisional margins. The latest NCCN Guidelines recommend a 4-6mm resection margin for lrSCC. It is considered infeasible to recommend the defined margin for hrSCC due to the wide variability of clinical characteristics. While the EDF-EADO-EORTC guidelines stated a minimal standardized margin of 5 mm for lrSCC and 6-10 mm for hrSCC (3, 4). It has also been suggested that histologic margins at or above 5 mm may increase survival in patients receiving WLE for advanced cSCC of the head and neck (24). The British Association of Dermatology recommends a 6-mm excision margin for hrSCC to achieve an oncologic clearance rate of 95% (8). For increased safety, Schell (5) found that 13.25 mm margins were required to achieve a 95% clearance rate in hrSCC, which is unreasonable for most patients. High-risk tumors often originate in the head and neck, where there are limited anatomic functions and high cosmetic standards, leading to disagreement over the appropriate surgical margin width. Mohs micrographic surgery (MMS) is widely regarded as the most effective treatment option. This method involves examining the specimen during surgery to ensure complete removal of the tumor while preserving normal tissue (6, 7). However, promoting MMS is challenging due to its time-consuming nature, high labor costs, equipment requirements, and the need for cooperation from dermatologists.

All patients recruited in the study were classified as high-risk or very-high-risk cSCC according to the NCCN Guidelines. Our results showed that the complete resection rate increased with increasing peripheral surgical margins, and the differences between the expansion cuts at 0 mm, 4 mm, and 8 mm were statistically significant in both groups of patients (p < 0.01). In the unbiopsy or incisional biopsy group, 20 cases (33.3%) remained unclear according to the dermoscopic boundary with a 4-mm expansion. The extent of resection to 8 mm affected tumor remnants, and the clearance rate grew to 98.3% and was statistically different from the 0-mm and 4-mm groups (p = 0.002 and p = 0.047). Only one person had a residual tumor with no recurrence after follow-up, probably due to artefacts in pathological sections. Poor quality sections can lead to the ambiguous observation of cell structure, or some artefacts may result in misdiagnosis. Notably, a positive margin of 8 mm was also seen at 4 mm in the same direction. This corresponds to a single focal, continuous tumor growth pattern where excessive excision of normal tissue is required to ensure complete removal. Hence MMS may be the most suitable solution. Marrazzo (25) reported outcomes in patients with hrSCC treated by MMS alone, finding 8.8% local recurrences or metastases, and 1.1% deaths. Our data indicated that 6 patients (10%) ended up with postoperative recurrences or metastasis, which is close to the rate of MMS. Five were aged >80 years old, having tumors over 2 cm in diameter and located around the ears or on the scalp. And another had leukemia with a history of suppressive immunotherapy. All these conditions can lead to deep tumor infiltration and a relatively high risk of recurrence. Therefore, we recommend an 8-mm margin under dermoscopic guidance, which removes 98.3% of the lesions and achieves a comparable prognosis to MMS.

Following an excisional biopsy, both tumors and some of the surrounding normal tissues were removed, resulting in the typical dermoscopic features were replaced by neovascularization, sutures, and scars. The tumor clearance rate after the 4-mm expansion was 93.3%, with only two positive pathological returns and no statistical difference between 8-mm expansion (100%). None of this group experienced a recurrence following the surgery, and even half of the re-excised patients showed no presence of tumor cells in the postoperative pathology report. This could be attributed to the fact that dermoscopy tends to confuse the tumor-like appearance caused by inflammation and dermal scarring. Despite achieving a satisfactory resection with a 4-mm dilation, the 100% tumor clearance rate of 8 mm is clinically preferable from a patient-benefit perspective. As reported by Gunson (26), tissue distortion caused by shrinkage and scars makes identifying the true margins unreliable. Tumor cells may metastasize between the original defect and adjacent tissue. Therefore, if both functional and aesthetic needs can be considered, we recommend an 8-mm expansion under dermoscopy to ensure the most favorable prognosis.

Based on our observations, none of the poor prognosis patients exhibited a positive resection margin at 8 mm, which may be related to the following factors: firstly, histopathologic sectioning that missed the target area with residuals due to sectioning limitations; secondly, there may be tumor remnants in the deep margin. Dermoscopic assessment of the lateral tumor border assessment is an alternative and adequate method, but insufficient for tumor depth. Our findings were similar to those of other studies, and deep tumor remnant after excisional biopsy is also problematic (26). After wound closure or reconstruction with transferred flap tissue, deep margins may be buried and residual tumors could exist anywhere at the base of the defect. Accordingly, postoperative monitoring of tumor resection margins and reevaluation or resection of suspected residual tumors are necessary.

## Conclusion

Dermoscopy was proven effective in identifying surgical margins with greater accuracy than visual inspection alone. Direct dermoscopy-guided WLE with at least 8-mm expansion was recommended for hrSCC. Dermoscopy also assisted in determining surgical margins at the healing biopsy site. A 4-mm dermoscopy-detected excision margin can successfully resect 93.3% of tumors, while an 8-mm excision margin can reach 100.0%, making 8 mm still the recommended range of expansion.

## Limitation

First, the follow-up period is insufficient to assess long-term recurrence. Out of our patients, 24 (26.7%) were tracked for less than three years but more than two years. No relapse occurred in these cases, which necessitates that we pay more attention to observing these patients during future follow-up. Second, the results obtained in this study need to be validated in a large-sample multicenter study. Third, the present study did not evaluate the deep marginal positive rate, and dermoscopy may miss deep-seated residual tumors.

## Data availability statement

The raw data supporting the conclusions of this article will be made available by the authors, without undue reservation.

## Ethics statement

The studies involving human participants were reviewed and approved by the Ethics Committee of the First Affiliated Hospital with Nanjing Medical University (IRB-GL1-AF05; 2021-NT-32). The patients/participants provided their written informed consent to participate in this study. Written informed consent was obtained from the individual(s) for the publication of any potentially identifiable images or data included in this article.

## Author contributions

DW: conceptualization, funding acquisition, and resources. ZL, SH, FL, XW, ML, HW, JJ, and YZ: investigation. ZL, SH, and FL: formal analysis. ZL and SH: writing—original draft preparation. ZL and SH: writing—review and editing. SH and DW: supervision. ZL and DW: project administration. All authors contributed to the article and approved the submitted version.
